# Population Density and Driving Factors of North China Leopards in Tie Qiao Shan Nature Reserve

**DOI:** 10.3390/ani11020429

**Published:** 2021-02-07

**Authors:** Mengyan Zhu, Muhammad Zaman, Meng Wang, Kasereka Vitekere, Jianzhang Ma, Guangshun Jiang

**Affiliations:** Feline Research Center of Chinese State Forestry and Grassland Administration, College of Wildlife and Protected Area, Northeast Forestry University, Harbin 150040, China; zhumengyan@outlook.com (M.Z.); zam.wlm@gmail.com (M.Z.); wang_meng@yeah.net (M.W.); kasvitekere@hotmail.fr (K.V.)

**Keywords:** North China leopard, camera trap, population size, environmental factors, anthropogenic factors

## Abstract

**Simple Summary:**

The North China leopard is a subspecies of leopard distributed in China, but little is known about its population status. This study selected the most active areas of North China leopards to determine the population density and distribution of North China leopards. We found that different prey had different effects on the density distribution of North China leopards. Environmental factors and human factors are also important factors affecting the population density distribution of North China leopards. These results provided an effective basis for the protection of North China leopard population and management evaluation of the reserve. It also provided effective methods for the protection and management of other endangered species.

**Abstract:**

The North China leopard (*Panthera pardus japonesis*) is a rare leopard subspecies distributed only in China. In this study, we conducted camera-trap surveys of a North China Leopard population in Tie Qiao Shan Nature Reserve, Shanxi Province, China. We estimated population abundance and density distribution, and explored the effects of distribution of different prey populations, habitat, and anthropogenic factors on the spatial distribution of North China leopard density. Our results suggested that the North China leopard density was 4.23 individuals/100 km^2^, and that 17.98 individuals might live within the study area. The population density of the North China leopard increased with the distribution of wild boars, and, on the contrary, decreased with the distribution of roe deer. We found that habitat environmental factors and anthropogenic interference also significantly affected the population density and spatial distribution of the North China leopard. These insights informed us that in order to protect this predator, which is only distributed in China, we should adopt a comprehensive customized adaptive landscape protection strategy.

## 1. Introduction

The leopard (*Panthera pardus*) is a solitary, reclusive species of big cat. It is also the most widespread felid, extending across much of Africa, as well as in Asia, from the Middle East to the Pacific Ocean [1]. Leopard habitat varies greatly, and they are found in tropical forests, grassland plains, deserts, and alpine areas [2]. Leopards are facing global habitat fragmentation and loss of suitable habitat [3]. The North China leopard (*Panthera pardus japonesis*) is the only leopard subspecies distributed only in China. The North China leopard is the flagship species of the forest ecosystem in North China and a symbol of ecological environmental quality. However, there have not been extensive studies on the population, density, distribution, endangered status, and habitat quality of North China leopards [4], which severely restricts the effective development of its population and habitat protection and management.

The habitat selection of endangered wild animals has always been the core concern of researchers and conservation managers [5]. Wild animals need to consider factors such as habitat quality, resource availability, intra-species competition, and inter-species interactions when choosing their habitat environment [6]. The availability of food is an important factor in the choice of habitat for predators [7]. Reduced food availability will directly lead to the death or even disappearance of predators [8]. Especially for large carnivores, it is more difficult to obtain food than herbivores due to their different predation patterns; therefore, having abundant prey plays a vital role in choosing the habitat and spatial distribution of carnivores [9].

The loss of living space or habitat is one of the main factors leading to the reduction or even extinction of endangered or threatened species populations [10]. Currently, the reduction and fragmentation of suitable habitats for species is a challenge for wildlife researchers and conservation managers worldwide [11]. In recent years, with the increase in population, the seriousness of conflicts between humans and wild animals has gradually become another challenge to protect wild animals [12]. Especially in a populous country such as China, human disturbance is one of the issues that cannot be ignored in the field of wildlife research and protection.

Most big cats are solitary, avoid human contact, and are most active at night [13]. In combination with low population density, these factors make traditional field survey methods difficult. In recent years, studies have shown that large carnivores are the most studied species using infrared camera trap technology [14]. Individual identification technology based on distinctive fur patterns makes identification of individual large mammals possible and accurate with camera trap photograph data [15]. This method has subsequently been widely used in the monitoring and research of big cats [16].

In this study, we selected the protected area with the most frequent North China leopard activities known thus far, yet there are always human activities and grazing behaviors in the protected area. We estimated the density and spatial distribution of the North China Leopard populations by using spatially explicit capture–recapture (SECR) models with camera trap data. Then, the generalized additive model was used to explore the influence of environmental factors and human factors on the population density and spatial distribution of North China leopards. We hypothesized that (1) the spatial distribution of different prey has different effects on the population density and spatial distribution of North China leopards; (2) elevation, slope, and river are important environmental factors affecting the density distribution of the North China leopard population; and (3) anthropogenic disturbance reduces population density distribution of North China leopards.

## 2. Materials and Methods

### 2.1. Study Area

**Figure 1:**
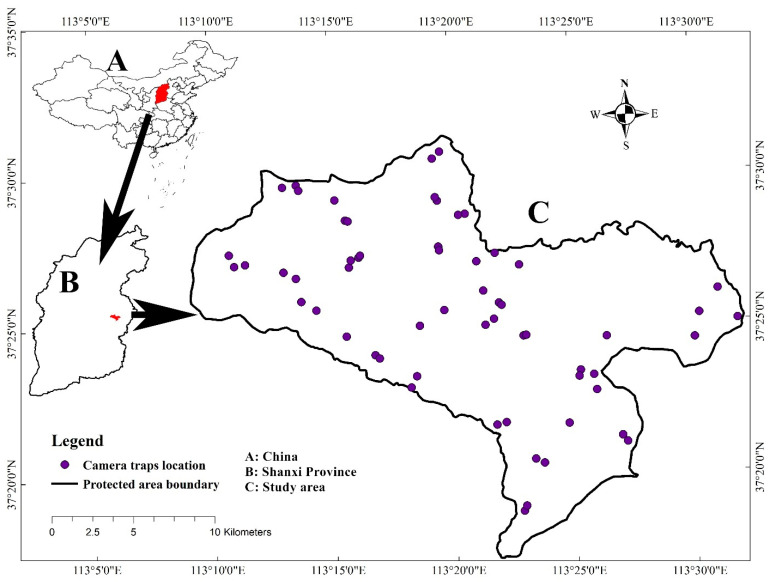
Illustration of study area and camera trap location. The study area is located in Tie Qiao Shan Nature Reserve, Shanxi Province, China.

The study area was located in the Tie Qiao Shan Nature Reserve, Shanxi Province, China (113°05′–113°35′ E, 37°13′–37°34′ N; Figure 1). The total area of the reserve is 353.52 km^2^. This reserve is the most active area in the entire North China leopard distribution area. The reserve contains 39 villages, and the government allows grazing in the reserve in order to increase the income of the villagers. The North China leopard population in the reserve is always facing the problem of human–wildlife conflict. The highest altitude is 1827 m, mostly between 1400 and 1700 m. The area has a temperate continental monsoon climate, of alpine and humid type, with an average annual temperature of 6.3 °C. The annual precipitation is 593 mm. Mainly concentrated in summer in July and August. Common North China leopard prey are mainly wild boar, roe deer, hare, and pheasant.

### 2.2. Camera Trap Erection

**Figure 2:**
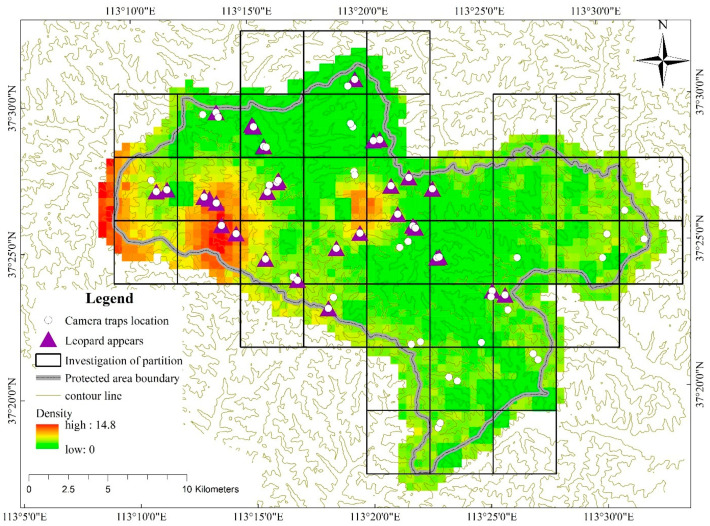
The spatial distribution of the North China leopard was predicated by the spatially explicit capture–recapture (SECR) model. The color gradient of each pixel represents the density gradient of North China leopard population at each pixel. Only pixels judged to be suitable habitat are included, and the size of each pixel is 0.25 km^2^.

Using GIS technology, we divided the survey sample area of 4 × 4 km^2^ into the whole territory of Shanxi Tie Qiao Shan Nature Reserve (Figure 2). After removing the marginal sample, we finally determined the survey sample to be 30 sample areas. In each survey sample, 2 infrared cameras (LTL-6210, ShenzhenWeikexin Science and Technology Development Co., Ltd., Shenzhen, China) were set facing each other at each trap station to increase capture probabilities and capture the fur patterns on both sides of the leopard [17]. At the same time, 1 or 2 cameras were randomly deployed in each survey sample area in an area with good vegetation environment to detect the occurrence information of North China leopard prey. Infrared camera data were retrieved every 3 months. We recorded the occurrence information and time of the species. A total of 102 cameras were actually installed (Figure 2). Due to the interference of human activities, we only obtained data from 77 cameras, of which 31 cameras captured images of the North China leopard. In terms of the inspection of camera data, after removing similar data from nearby camera traps, we ultimately used 60 camera trap data in this study. The inspection time was from March 2017 to May 2019.

### 2.3. Data Analysis

We used Extract Compare software to identify the photos of individual North China leopards, with the location and time of initial capture being taken into account [15]. Extract Compare is a software that uses three-dimensional technology to extract patterns on the body side of the target animal in the image data and identify the target animal by the similarity of the pattern on the body side [15].

Then, leopard density was estimated using information on capture histories in combination with the spatial locations of captures under a unified Bayesian modeling framework.

The SECR model was a logistic regression model for binary observations of individuals captured at a particular camera trap during sampling occasions [18]. It not only included individual heterogeneity in capture probabilities, but also offered estimates of spatial variation in animal densities [19]. We used this model implemented in SPACECAP, a user-friendly R software package, to estimate the densities of the leopard population (i.e., number of leopards per 100 km^2^) using all the collected camera trap data [19]. We established a 1 km buffer zone along the boundary of the reserve to ensure that the reserve was closed and all marginal areas were considered. The buffered area was used to estimate the population density of North China leopards.

RAI is the relative abundance index of the species [20]. The calculation method of relative abundance index is

RAI = (Ni/TRAPDAYi) × 100(1)

The Ni is the number of independent valid photos of species i, which means that adjacent photos of the same species with an interval of no more than 30 min are the same independent valid event. The TRAPDAYi is the camera working day [21]. Medium-sized ungulates occupy the most important part of the leopard’s diet. Considering that wild boar and roe deer are the only medium-sized prey in North China in the study area, this study only calculated the relative abundance index of wild boar (*Sus scrofa*) and roe deer (*Capreolus capreolus*) in each camera location.

Through the generalized linear regression model, we determined the relationship between the relative abundance index of wild boar and roe deer in the sample square, as well as environmental factors and human interference, and then used this to predict the spatial distribution of wild boar and roe deer in the entire study area. Environmental variables and human interference were extracted through ArcGIS 10.6. The environmental and human data used in this study are shown in Table 1.

Lastly, we used generalized additive models (GAMs) to determine relationships between roe deer distribution, wild boar distribution, and leopard density [22]. We used Spatial Analysis in Macroecology (SAM) software to check the spatial auto-correlation in the residuals of GAMs, and then ran the order of “kriging” in Arcmap to realize the spatial interpolation to ensure that the spatial auto-correlation was mitigated [20].

## 3. Results

### 3.1. Population Density and Spatial Distribution of North China Leopard

From March 2017 to May 2019, a total of 102 cameras were installed, of which 77 cameras received valid data and 31 cameras captured images of the North China leopard. A total of 12 North China leopards were successfully identified. The parameters of SECR models for the North China leopard camera trap data based on 12 observed individuals are summarized in Table 2. The recapturing model estimates that the population density of North China leopard in the study area was 4.23 (2.82–5.64) per 100 km^2^. The total number of North China leopard individuals in the study area was 12–24 (approximately 17.98). The spatially explicit leopard density distribution map was created as a raster image with a resolution of 0.25 km^2^ per grid cell or pixel (Figure 2).

### 3.2. The Relative Abundance Index and Distribution of Wild Boar and Roe Deer

The average relative abundance index of roe deer in the study area was 5.23. The model fitting results show that the distribution of the roe deer was positively correlated with elevation, aspect, and distance to the village; and negatively correlated with the slope, road, and net photosynthesis productivity. The average relative abundance index of wild boar was 8.97. Unlike roe deer, the distribution of wild boar was positively correlated with elevation, rivers, mixed forests, and woody savanna; and negatively correlated with slope, villages, distance to roads, and net photosynthesis productivity (Supplementary Data A–C).

### 3.3. The Relationship between the Spatial Distribution of North China Leopard Density and Its Prey and Other Environmental Factors

**Figure 3:**
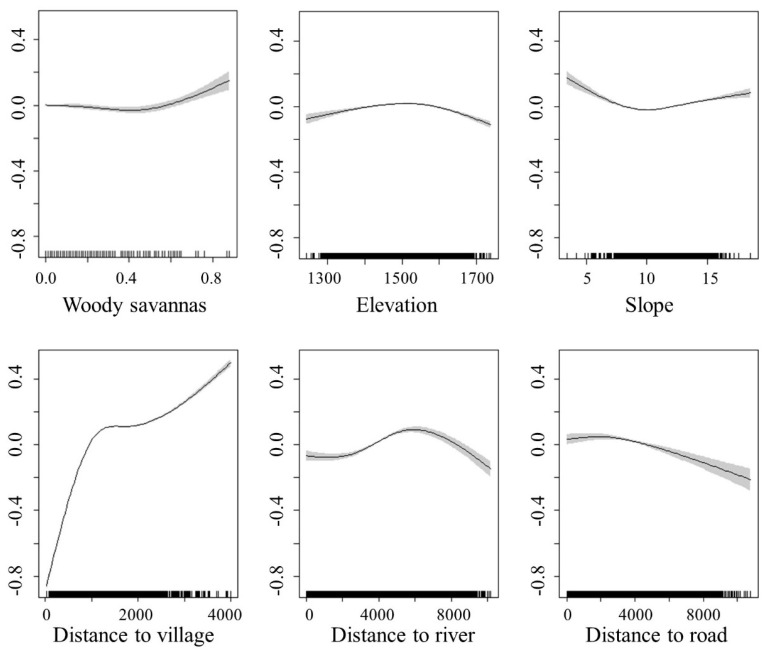
Relationships between the North China leopard density (individuals/0.25 km^2^) and environmental factors by generalized additive model (GAM). The ordinate shows the estimated population density of North China leopard (individuals/0.25 km^2^), and the abscissa shows environmental and human factors.

The model fitting results showed that the North China leopard preferred to appear at an altitude of about 1500 m and a distance of 6 km from the river. The North China leopard avoided gentle slope terrain. It started to appear after 1 km from the village and moved frequently as the distance between the village increased. The density of North China leopards is positively correlated with the area of woody savanna, and negatively correlated with the distance from the road (Figure 3).

**Figure 4:**
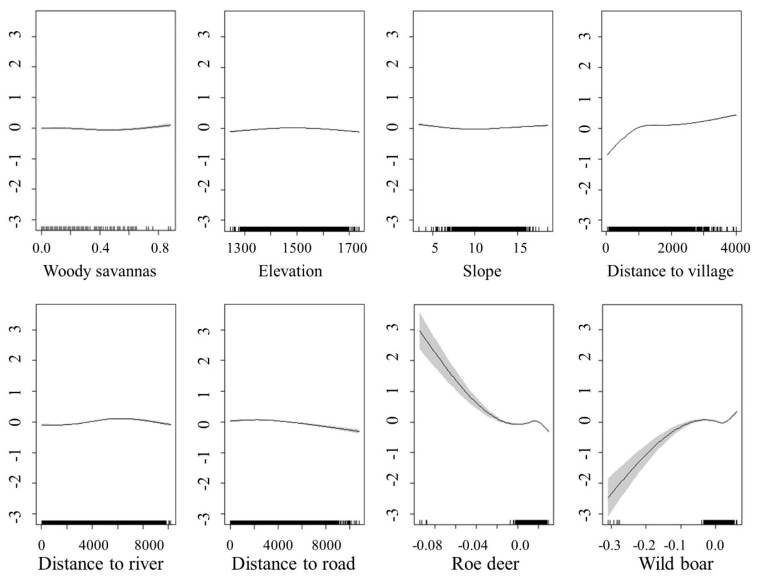
Relationships between the North China leopard density (individuals/0.25 km^2^) and two kinds of prey and environmental factors by generalized additive model (GAM). The ordinate shows the estimated population density of North China leopards (individuals/0.25 km^2^), and the abscissa shows the relative abundance of prey, and environmental and human factors.

The population density of North China leopards increased with the increase of the frequency of wild boar, and on the contrary, the population density of North China leopards decreased with the increase of the frequency of roe deer (Figure 4).

## 4. Discussion

### 4.1. Population Size and Density Distribution of North China Leopard

As a big cat, the population size and population density of the North China leopard in the study area are relatively high. This result is much higher than the density of Amur leopards distributed in eastern China [23]. There are reports that the habitat of the North China leopard is facing widespread shrinkage and destruction [1]. However, the population density of North China leopards in the study area is relatively high [24]. In addition, according to the unpublished gene exchange study of the North China leopard population in the research group, research found that the North China leopard in the study area had serious inbreeding pressure. Therefore, the severe destruction of the North China leopard habitat in the study area and the reduction of suitable habitat have caused the North China leopard population to be compressed in a small area, resulting in a high population density of the North China leopard.

Two of the core distribution areas of the North China leopard population in the study area are not in the central area of the reserve, which shows that the planning of the reserve restricts the effective protection of the North China leopard. This may be because the reserve was originally established to protect the Chinese pine forest, and wild animals preferred to appear on the edge of the forest [25]. At the same time, big cats tend to avoid dense forests due to their size [13]; therefore, the dense Chinese pine forest in the core area of the reserve is not suitable for the distribution of North China leopards. As an umbrella species of large carnivores, the protection of the population of large carnivores plays an important role in the restoration of the entire ecosystem in the distribution area [26]. At present, many protected areas are not established to protect top predators. People pay more attention to wildlife protection, and thus the functional planning of the protected areas should be adjusted in time to deal with the new protection situation [27].

### 4.2. The Relationship between the Distribution of North China Leopard Prey and the Population Density Distribution of North China Leopard

There is no obvious complementary relationship between the distribution of wild boar and roe deer in the study area, indicating that the competition between the two prey is not obvious (Supplementary Data B and C). The North China leopard’s preference for wild boar is an active choice rather than a passive choice. Among the North China leopard prey in the study area, the relative abundance index of wild boar was found to be higher than that of roe deer, indicating that the distribution of wild boar in this area is more common. This may be due to the large area of Chinese pine forest in the study area [28]. Acevedo et al. found that wild boar prefer real broad-leaved mixed forest as habitat, which is consistent with the results of this study [29]. At the same time, some studies believe that the emergence of wild boar will also cause some interference to other ungulates, which may also explain why the relative abundance index of wild boar is higher than that of roe deer [30]. It is found that villages and roads have effects on wild boars and roe deer, but the effects are the opposite. This may be due to the fact that wild boars are more likely to be distributed on the edge of the forest and even feed on crops, while roe deer are more afraid of people and avoid human interference [28].

### 4.3. The Relationship between Environmental Factors and Human Factors and the Population Density Distribution of North China Leopards

Leopards strongly avoid areas that may encounter humans during the day, and choose these areas to varying degrees at night [31], which explains why the population density of North China leopards gradually increases after moving away from the village. An analysis of the diet of leopards in a Congolese forest found that they feed on smaller animals in areas close to villages, while leopards farther away will also prey on larger animals; this is related to the spatial distribution of leopards and the distance from the village [32].

Elevation plays a vital role in the distribution of big cats [33]. In this study, the North China leopard was found to be located in a higher altitude area, and the index density was highest in the 1500 m area; the high elevation distribution may be due to the squeezing of the range of human activities. Similarly, snow leopards are extremely sensitive to altitude, and as the altitude increases, the habitat utilization rate gradually increases [34]. The correlation between North China leopards and altitude is not linear, and they tend to choose the middle elevation area in the study area. This is generally the area with the best habitat quality that can provide sufficient prey and living space [35]. At the same time, the North China leopard chooses to avoid medium slopes and choose flat or steep slopes. The gentle slope areas are the main grazing areas. For a long time, the North China leopards avoided human activities and interference from grazing behavior.

Water source is one of the main factors for the existence of wild animals [36], and North China leopards prefer to appear in areas about 6 km away from the river. In the mountainous landscape, the habitat utilization of clouded leopards is obviously negatively correlated with river distance [37]. In the study of three endangered cats (Sunda clouded leopard, Asiatic golden cat, and marbled cat) in Sumatra, research found that the three cats all had a strong correlation with the river distance, but the difference was such that the three cats had corresponding differences in the river distance [38]. It can be seen that different types of cats in different regions have different responses to river distances, but this also illustrates the importance of rivers to their distribution. In-depth research on the specific relationship between the density distribution of North China leopards and the distance of the river is needed to reveal why the distribution of North China leopards prefer the area 6 km away from the river.

Forest coverage and type will affect the habitat utilization and spatial distribution of cats [2]. The larger the area of woody savanna, the higher the population density of North China leopards. This may be due to the higher distribution of the main prey of North China leopards in this area.

## 5. Conclusions

Prey is the primary condition for the recovery of the North China leopard population. A reasonable prey community structure and stable prey population density are important guarantees for the smooth progress of the North China leopard protection work. More attention should be given to the North China leopard protection process. It is necessary to better restore the population of North China leopards by controlling the population of wild boar and roe deer in nature reserves; for example, increasing artificial feeding points in areas far from human activities and areas where wild boar activities are intensive. Reducing the number of villages and the intensity of human activities in the reserve is conducive to the protection and restoration of the North China leopard population. Elevation, slope, and distance to river are important factors for the population density and spatial distribution of North China leopards. In order to protect the North China leopard, a large cat that is unique to North China, it is necessary to comprehensively consider various environmental factors and formulate practical protection and management strategies according to the landscape scale. Finally, the study provides a good example for revealing the spatial relationship between North China leopards and their prey and other environmental variables, thereby providing a good example for formulating reasonable habitat protection and management strategies.

## Figures and Tables

**Table 1:** Environmental variables used to analyze the density distribution of North China leopards and the distribution of their prey. Note: Data were processed in ArcGIS 10.6 in grid format (0.5 × 0.5 km pixel) on the basis of re-sampled or interpolated measurements. Data were extracted from the National Geomatics Center of China (http://www.ngcc.cn/ngcc/ (accessed on 30 June 2018)).

Variable	Description of the Habitat Factor	Data Type	Unit
Elevation	30 m resolution DEM conversion 0.5 km resolution elevation grid	Continuous	m
Slope	30 m resolution DEM conversion 0.5 km resolution slope grid	Continuous	m
Aspect	30 m resolution DEM conversion 0.5 km resolution aspect grid	Continuous	m
Distance to village	Distance from the central point of each pixel to the nearest village	Continuous	m
Distance to road	Distance from the central point of each pixel to the nearest road	Continuous	m
Distance to river	Distance from the central point of each pixel to the nearest river	Continuous	m
Net production	Difference between how much carbon dioxide is taken in by plants compared to how much is put out by them; this difference is total amount of carbon dioxide taken in by plants, called net primary productivity; extract in raster layer	Continuous	gCm^−2^ yr^−1^
Mixed forest	Mixed forest area ratio of each pixel	Continuous	Ratio
Woody savanna	Woody savanna area ratio of each pixel	Continuous	Ratio

**Table 2:** Posterior summaries of the parameters of SECR model for the North China leopard camera trapping data in terms of the 12 observed individuals. Note: N is the number of North China leopard activity centers in the population exposed to sampling, D is the density per 100 km^2^, ψ is the data augmentation parameters, σ is the parameter in the bivariate normal pdf, β is regression coefficient that measures the behavioral response, and p0 is detection probability; the estimates of p1 and p2 are also reported. p1 is the encounter probability for individuals that have not previously been encountered, and p2 is the encounter probability for individuals subsequent to their initial encounter.

Parameter	Mean	SD	5%	95%
*ψ*	4782.32	1689.66	3133.31	6741.81
*σ*	0.03	0.01	0.01	0.04
*β*	1.38	0.38	0.69	2.18
*p*0	0.43	0.11	0.25	0.66
N	17.98	3.45	12	24
*D*	4.23	0.81	2.82	5.64
p1	0.03	0.01	0.01	0.04
p2	0.01	0.02	0.06	0.13

## Data Availability

The data presented in this study are available upon request from the corresponding author.

## Supplementary Materials

The following are available online at https://www.mdpi.com/2076-2615/11/2/429/s1: Supplementary Data A: Summary of the generalized linear model parameters. Supplementary Data B: The relative abundance index of wild boar per 0.25 km^2^ by generalized linear model prediction. Supplementary Data C: The relative abundance index of roe deer per 0.25 km^2^ by generalized linear model prediction.
