# Supplementary material for: Population Density and Driving Factors of North China Leopards in Tie Qiao Shan Nature Reserve
Source: Animals (Basel). 2021 Feb 7;11(2):429. doi: 10.3390/ani11020429 (PMC7915284; doi:10.3390/ani11020429)

Supplementary data A. Summary of the generalized linear model parameters. Select the relative abundance index of camera trap wild boar and roe deer as the dependent variable.

	Parameter	Estimate	S.E.	T value	P value
Wild boar	Intercept	6.845	1.083	6.32	<0.001
	Elevation	0.002	<0.001	2.41	0.02
	Slope	-0.22	0.026	-8.52	<0.001
	Distance to village	<-0.001	<0.001	-3.54	<0.001
	Distance to road	<-0.001	<0.001	-9.98	<0.001
	Distance to river	<0.001	<0.001	2.95	0.003
	Net production	<-0.001	<0.001	-9.13	<0.001
	Mixed forest	0.43	0.17	2.50	0.012
	Woody savannas	2.75	0.61	4.52	<0.001
Roe deer	Intercept	-0.29	1.18	2.726	0.81
	Elevation	0.002	<0.001	2.726	0.006
	Slope	-0.06	0.03	-1.765	0.07
	Aspect	0.006	0.001	4.83	<0.001
	Distance to village	<0.001	<0.001	1.95	0.05
	Distance to road	<-0.001	<0.001	-3.255	<0.001
	Distance to river	<-0.001	<0.001	-3.936	<0.001

Supplementary data B. The relative abundance index of wild boar per 0.25 km^2 by generalized linear model prediction.

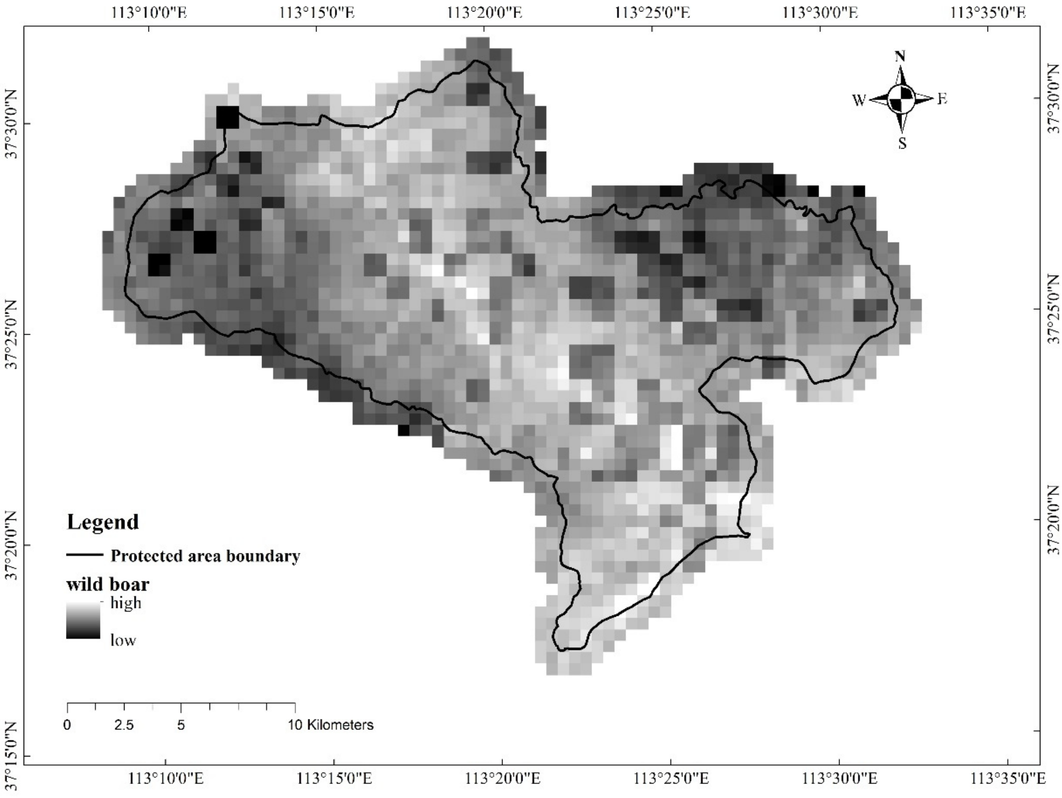

Supplementary data C. The relative abundance index of roe deer per 0.25 km^2 by generalized linear model prediction.

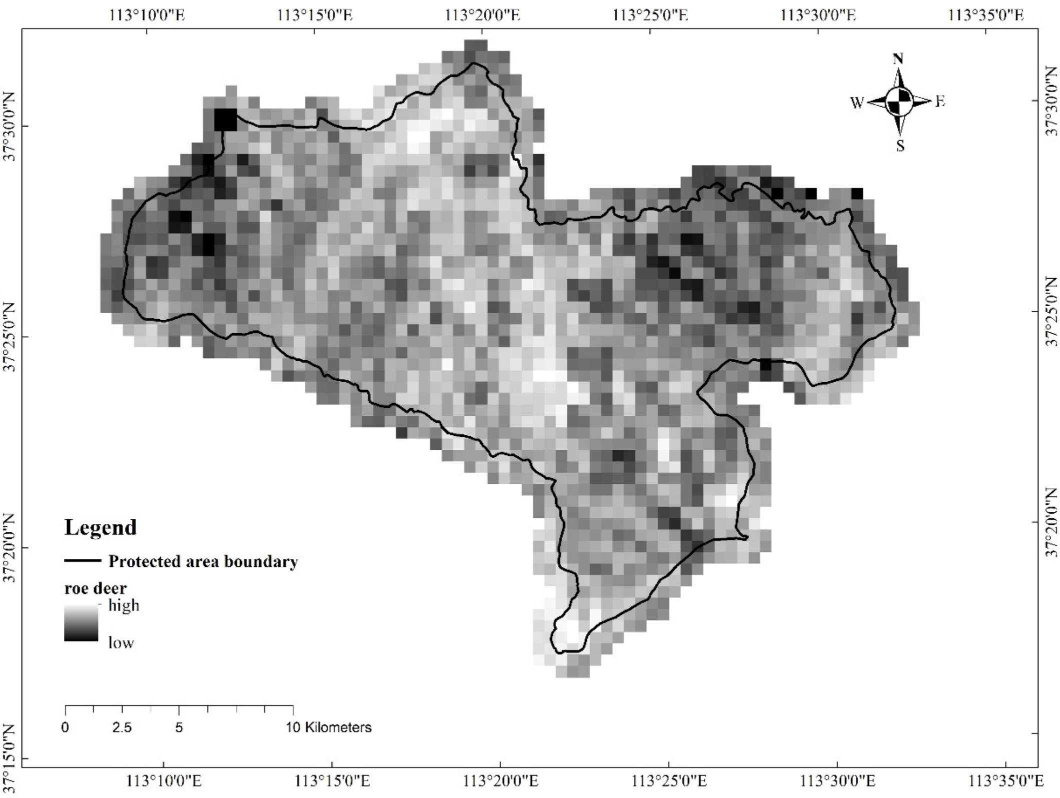

Supplement: Supplementary file 1 [file animals-11-00429-s001.pdf]
